# Effects of automated insulin delivery systems on glucose control in subgroups of adults with type 1 diabetes in clinical practice over 2 years in Sweden

**DOI:** 10.1038/s41598-026-37158-x

**Published:** 2026-03-31

**Authors:** Ramanjit Singh, Henrik Imberg, Shilan Seyed Ahmadi, Sara Hallström, Johan Jendle, Bengt-Olov Tengmark, Anna Folino, Marie Ekström, Marcus Lind

**Affiliations:** 1 Department of Molecular and Clinical Medicine, Sahlgrenska Academy, University of Gothenburg, Gothenburg, Sweden; 2 Department of Medicine, Geriatrics and Emergency Care, Sahlgrenska University Hospital/Östra Hospital, Region Västra Götaland, Sweden; 3 Statistiska Konsultgruppen Sweden, Gothenburg, Sweden; 4 School of Medical Science, Faculty of Medicine and Health, Örebro University, Örebro, Sweden; 5 Citydiabetes, Stockholm, Sweden; 6 Department of Medicine and Emergency, Sahlgrenska University Hospital/Mölndal Hospital, Gothenburg, Sweden; 7 Department of Medicine, NU Hospital Group, Uddevalla, Sweden

**Keywords:** Type 1 diabetes, Time in range, Automated insulin delivery, Hybrid closed loop, Diseases, Endocrinology, Health care, Medical research

## Abstract

Several studies have demonstrated improvements in glucose control after initiation of automated insulin delivery (AID). This study aimed to evaluate glycemic outcomes and treatment-related barriers in subgroups of adults with type 1 diabetes (T1D) using AID systems in routine clinical practice. We analyzed data from 142 adults with T1D using Tandem Control IQ or MiniMed 780G. Correlation analyses were conducted to evaluate how changes in glycemic outcomes varied in relation to baseline characteristics. Mean age was 42 years, 40.8% were females, and median follow-up time was 1.7 years. Individuals with higher hemoglobin A1c (HbA1c) had less time below range (TBR) before starting AID (*P* < 0.001). Effects on glucose control were similar irrespective of sex, prior insulin therapy, body mass index, and diabetes duration, but varied in relation to glucose control. Following AID initiation, the estimated reduction in HbA1c was 1.3% (14.3 mmol/mol) in individuals with a baseline HbA1c of 8.6% (70 mmol/mol), compared to 0.3% (2.9 mmol/mol) in those with a baseline HbA1c of 6.7% (50 mmol/mol) (*P* < 0.001). Among individuals with a baseline TIR of 50% and 70%, the estimated increase in TIR following AID initiation was 19.0% and 7.2%, respectively (*P* < 0.001). Greater reductions in HbA1c were associated with smaller reductions in TBR and vice versa (*r* = − 0.29, *P* = 0.003). Among MiniMed 780G users, older age correlated with greater improvements in TIR (*P* = 0.005). Skin reactions tended to be more frequent in women (41%) than men (26%), *P* = 0.057. In conclusion, AID system use was associated with substantial reductions in HbA1c in individuals with poor glycemic control. Clinically meaningful reductions in HbA1c were achieved also in individuals with an HbA1c closer to target who additionally gained greater reductions in TBR.

**Supplementary Information:**

The online version contains supplementary material available at 10.1038/s41598-026-37158-x.

## Introduction

The use of continuous glucose monitoring (CGM) and continuous subcutaneous insulin infusion (CSII) has increased markedly during the past few years. Despite advancements in diabetes technologies, only about 35% of adults with type 1 diabetes (T1D) in Sweden obtain target hemoglobin A1c (HbA1c) of < 7.0% (< 53 mmol/mol)^1^.

Automated insulin delivery (AID) systems combine CSII and real-time CGM with closed-loop algorithms which allow for automated basal insulin delivery, bolus corrections, and adjustable glucose targets. In randomized trials^2,3^, AID systems have demonstrated greater improvements in time in range (TIR; 70–180 mg/dL, 3.9–10.0 mmol/L) in both youth and adults with T1D when compared to sensor-augmented pump (SAP) therapy. In parallel, our knowledge of real-world effects of AID systems has expanded with several studies indicating high levels of efficacy and safety^4–10^.

However, not all AID users achieve the recommended glycemic targets of HbA1c < 7.0% (< 53 mmol/mol) and TIR > 70%. It is therefore important to better understand how treatment effects vary across clinical subgroups. In one large real-world study by Castañeda et al.^11^, including over 12,000 users of the MiniMed 780G system, lower TIR levels at baseline correlated with greater improvements in TIR post AID initiation. Similarly, Messer and colleagues^6^ found that children and young adults with higher baseline HbA1c achieved greater improvements in TIR after initiating AID. Older age has been associated with greater improvements in TIR in some studies^11,12^.

To our knowledge, no prior studies have included adults with both the Tandem Control IQ and Minimed 780G system in subgroup analyses and studies are overall lacking in Nordic countries. Furthermore, patient-reported outcomes such as barriers in daily use are most often not included. We recently published the results from an observational study^13^ evaluating the effects on glucose control, safety and treatment satisfaction in adults with type 1 diabetes using Tandem Control IQ or MiniMed 780G, the two most commonly used AID systems in clinical practice in Sweden. Following AID initiation, mean TIR increased by 14.5% within the first few months. Approximately one third of the study population reported skin reactions related to AID use. Building on previous findings, we aimed to identify baseline characteristics associated with greater glycemic improvements, as well as treatment-related barriers, following AID system initiation in routine clinical practice.

## Methods

This was a post hoc analysis of a previous observational study conducted at six outpatient diabetes clinics in Sweden: Sahlgrenska University Hospital/Östra and Sahlgrenska University Hospital/Mölndal (Gothenburg), NU-Hospital Group (Trollhättan–Uddevalla), Lidköping Hospital, Kungälv Hospital, and CityDiabetes Serafen (Stockholm)^13^. In brief, individuals with current or discontinued use of AID were identified through locally used quality registers at each clinic and subsequently invited to participate through random selection. Inclusion criteria were a diagnosis of T1D, current or discontinued treatment with either the MiniMed 780G or the Tandem Control IQ system, and age ≥ 18 years. Individuals were excluded if they had a diagnosis of type 2 diabetes, prior treatment with the MiniMed 670G system, diabetes duration less than one year, or pregnancy within six months before or during AID use. The study was conducted in accordance with the Declaration of Helsinki and was approved by the Swedish Ethical Review Authority (diary number 2023-00651-02). All participants provided written informed consent.

Annual HbA1c values (± 6 months) for the five years preceding AID initiation were collected retrospectively to capture annual variation while ensuring sufficient data availability. Additional quarterly HbA1c values and CGM data from the year prior to the initiation of AID along with the closest available HbA1c value and CGM measurements prior to start of AID were retrieved. Following AID initiation, HbA1c values and CGM data were collected at one month and at quarterly intervals thereafter. Four weeks of CGM data were retrieved at each time point, except for one month after the start of AID, where only two weeks of data were downloaded.

The primary endpoint was the change in TIR between the last available measurement before and after AID initiation^13^. Secondary endpoints included the change in HbA1c, change in time above range level 2 (> 250 mg/dL; >13.9 mmol/L), time below range level 2 (< 54 mg/dL; <3.0 mmol/L), and change in glycemic variability measured by the standard deviation (SD) of glucose values using the same pre- and post-initiation time points.

The study also included a questionnaire developed by the study group consisting of diabetes nurses and physicians with extensive clinical experience in the management of type 1 diabetes. The questionnaire aimed to identify practical barriers in the management of AID such as sensor problems, self-perceived knowledge of pump features and insulin leakage. Adverse events collected through the questionnaire included severe hypoglycemia (defined as hypoglycemia requiring assistance and/or leading to unconsciousness), ketoacidosis (defined as a pH value < 7.3 in combination with plasma glucose level > 250 mg/dL (> 14.0 mmol/L), blood ketones > 54 mg/dL (>3.0 mmol/L), and typical symptoms) and skin reactions due to adhesives used in sensor- or infusion sets. Self-reported events of severe hypoglycemia and ketoacidosis were verified through medical records. The questionnaire was answered by the participants after providing informed consent.

In this post hoc analysis, we evaluated how the effects on CGM metrics and HbA1c following AID initiation varied in relation to the following baseline characteristics; age, sex, diabetes duration, prior insulin administration route (multiple daily injections [MDI], or continuous subcutaneous insulin infusion [CSII]), smoking status, body mass index (BMI), TIR, time below range (TBR; <70 mg/dL, < 3.9 mmol/L), time above range (TAR; >180 mg/dL, > 10.0 mmol/L), mean glucose level, and glycemic variability measured by the SD of glucose values and coefficient of variation (CV). For the current analysis, changes were assessed using only the last measurement before AID initiation and the last available follow-up measurement. We also evaluated if skin reactions appearing in connection with AID system use were more common in certain subgroups defined by sex, age, diabetes duration, BMI, smoking status, prior insulin delivery method and baseline glycemic measures.

### Statistical methods

Descriptive data were summarized using means with SDs or medians with interquartile ranges (IQRs) for continuous variables, as appropriate, and counts with percentages for categorical variables. Changes in glycemic outcomes (HbA1c, TIR, TBR, TAR, mean glucose, and glycemic variability) before and after AID initiation were assessed using paired t-tests. All statistical analyses were performed using available cases.

Correlation analyses were conducted using Pearson correlation coefficients (r) to examine associations between baseline characteristics and changes in glycemic outcomes following AID initiation. Interaction analyses by AID system were subsequently performed to evaluate whether these associations differed between users of the MiniMed 780G and Tandem Control IQ systems. Mean changes in HbA1c, TIR, and TBR in relation to baseline values were estimated using linear regression. Comparisons between groups defined by sex, smoking status, and prior insulin delivery method were performed using two-sample *t*-tests in unadjusted analyses and analysis of covariance (ANCOVA) in adjusted analyses. Group comparisons and correlation analyses were conducted both unadjusted and with adjustment for age, sex, BMI, and diabetes duration. Model assumptions, including linearity, homoscedasticity, and normality of residuals, were assessed graphically using residual diagnostics and were found to be approximately fulfilled.

Associations between baseline characteristics and the reporting of skin reactions were analyzed using univariable logistic regression models, with odds ratios (ORs) and 95% CIs estimated per unit increase in the explanatory variable. Confidence intervals for differences in proportions were calculated using the Farrington–Manning score method.

All tests were two-tailed, with a significance level of 0.05. Given the exploratory nature of the analysis, no correction for multiple testing was applied. All analyses were conducted using SAS/STAT Software, version 9.4 (SAS Institute Inc., Cary, NC, USA).

## Results

A total of 142 individuals were recruited between October 2022 and February 2023. Baseline characteristics are presented in Table 1. The mean (SD) age was 42 (14) years, 58 (40.8%) were female, and 92 (64.8%) used the Tandem Control IQ system. A total of 104 (73.2%) participants used CSII before switching to AID. Median (IQR) follow-up time was 1.7 (1.1–2.6) years.

**Table 1:** Baseline characteristics of the study population, overall and by AID system.

	Total (*n* = 142)	Tandem Control IQ (*n* = 92)	MiniMed 780G (*n* = 50)
Age (years)	41.9 [14.2]	40.4 [14.0]	44.7 [14.1]
Female sex, n (%)	58 (40.8)	41 (44.6)	17 (34.0)
Diabetes duration (years)	23.3 [12.3]	21.5 [11.8]	26.6 [12.7]
Time in range (70–180 mg/dL; 3.9–10.0 mmol/L), (%)	56.4 [17.3]	53.1 [17.6]	62.6 [15.2]
HbA1c (%)	7.3 [1.1]	7.4 [1.2]	7.2 [0.9]
HbA1c (mmol/mol)	56.6 [11.7]	57.2 [12.7]	55.7 [9.8]
Smoking status, n (%)			
Current	6 (4.5)	2 (2.3)	4 (8.3)
Former	24 (17.9)	17 (19.8)	7 (14.6)
Never	104 (77.6)	67 (77.9)	37 (77.1)
BMI (kg/m^2^)	27.5 [4.5]	27.3 [4.8]	27.8 [4.0]
Macroalbuminuria, n (%)	4 (3.0)	1 (1.2)	3 (6.4)
Microalbuminuria, n (%)	5 (3.8)	4 (4.7)	1 (2.1)
eGFR (mL/min/1.73 m²)	106 [96–117]	111 [98–119]	102 [89–111]
Retinopathy, n (%)			
Mild	49 (36.3)	32 (37.2)	17 (34.7)
Moderate	19 (14.1)	11 (12.8)	8 (16.3)
Severe	22 (16.3)	9 (10.5)	13 (26.5)
Previous MI, CABG, or PCI, n (%)	4 (3.0)	1 (1.2)	3 (6.3)
Previous amputation, n (%)	2 (1.5)	1 (1.2)	1 (2.1)
Treatment prior to AID, n (%)			
MDI	38 (26.8)	31 (33.7)	7 (14.0)
CSII	104 (73.2)	61 (66.3)	43 (86.0)
CGM system prior to AID, n (%)			
Dexcom G6	90 (65.2)	89 (98.9)	1 (2.1)
Guardian 3	19 (13.8)	0 (0.0)	19 (39.6)
Guardian 4	29 (21.0)	1 (1.1)	28 (58.3)

Continuous variables are reported as mean [standard deviation] or median [IQR], as appropriate. Categorical variables are reported as number (percentage).

BMI, body mass index; CABG, coronary artery bypass graft; CSII, continuous subcutaneous insulin infusion; eGFR, estimated glomerular filtration rate; HbA1c, hemoglobin A1c; IQR, interquartile range; MDI, multiple daily injections; MI, myocardial infarction; PCI, percutaneous coronary intervention.

### Factors associated with improvement in glycemic outcomes

**Fig. 1:**
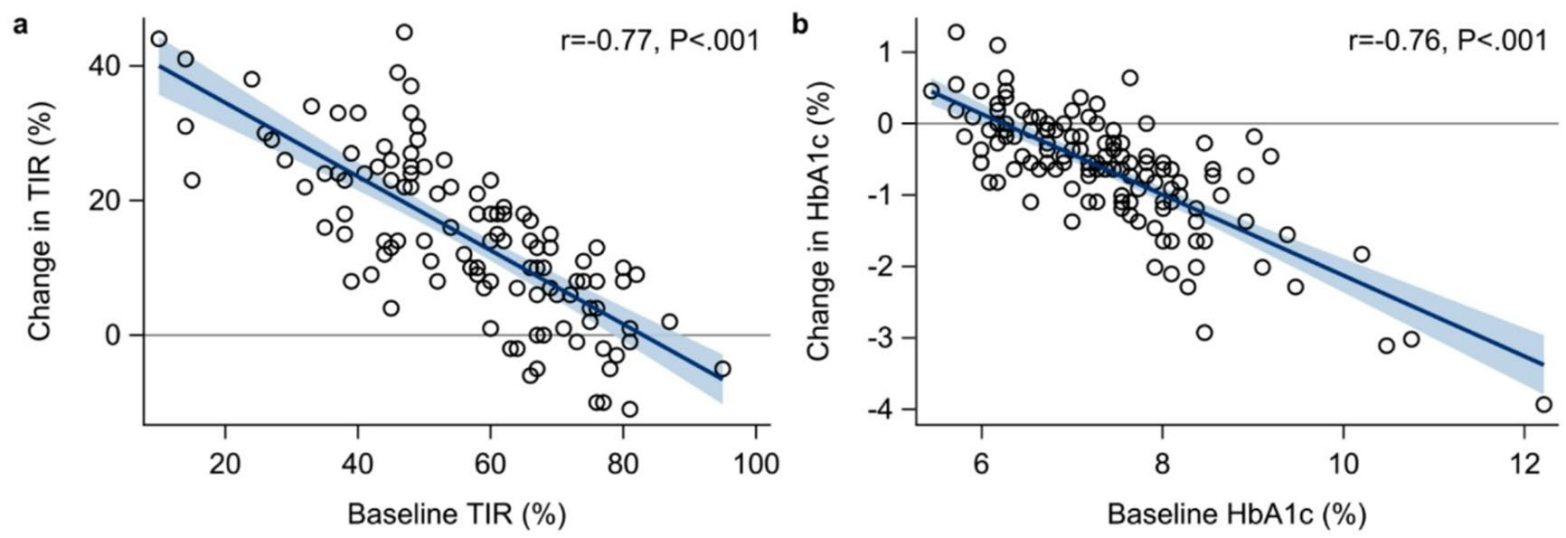
Association between baseline glycemic control and subsequent changes after initiation of automated insulin delivery (AID). (**a**) Change in time in range (TIR; 70–180 mg/dL, 3.9–10.0 mmol/L*)* versus baseline TIR. (**b**) Change in HbA1c versus baseline HbA1c. Points represent observed values. The blue line shows the fitted linear regression line, and the shaded band the corresponding 95% confidence interval for the trend line. r is the Pearson correlation coefficient.

Lower baseline TIR was strongly associated with greater improvements in TIR following AID initiation (*r* = − 0.77 [95% CI − 0.83, − 0.68], *P* < 0.001; Fig. 1a). A baseline TIR of 50%, 60%, and 70% was associated with a mean increase in TIR of 19.0% (95% CI 16.9, 21.1), 13.5% (11.5, 15.6) and 7.2% (5.2, 9.1), respectively (all *P* < 0.001). Lower TIR at baseline also correlated with greater reductions in TAR, HbA1c, mean glucose levels, and glucose variability measured with SD (all *P* < 0.001; Table 2).

**Table 2:** Correlation between baseline characteristics and subsequent changes in glycemic outcomes after AID initiation.

	Baseline TIR	Diabetes duration	BMI
Change in TIR	−0.77 (− 0.83, − 0.68) *P* < 0.001	0.02 (− 0.17, 0.20) *P* = 0.85	0.09 (− 0.11, 0.29) *P* = 0.38
Change in HbA1c	0.60 (0.45, 0.71) *P* < 0.001	0.12 (− 0.05, 0.29) *P* = 0.16	0.02 (− 0.17, 0.21) *P* = 0.82
Change in TAR	0.73 (0.63, 0.81) *P* < 0.001	−0.06 (− 0.25, 0.14) *P* = 0.54	−0.15 (− 0.35, 0.07) *P* = 0.17
Change in TBR	−0.05 (− 0.24, 0.14) *P* = 0.62	0.17 (− 0.02, 0.35) *P* = 0.078	0.07 (− 0.14, 0.28) *P* = 0.62
Change in mean glucose	0.69 (0.58, 0.78) *P* < 0.001	−0.03 (− 0.21, 0.15) *P* = 0.74	−0.18 (− 0.37, 0.02) *P* = 0.079
Change in SD of glucose values	0.37 (0.18, 0.53) *P* < 0.001	0.06 (− 0.14, 0.25) *P* = 0.57	−0.21 (− 0.40, 0.01) *P* = 0.060
Change in CV of glucose values	−0.21 (− 0.44, 0.05) *P* = 0.11	0.02 (− 0.24, 0.28) *P* = 0.88	−0.16 (− 0.43, 0.14) *P* = 0.30

Values are Pearson correlation coefficients (95% CI).

BMI, body mass index; CV, coefficient of variation; HbA1c, hemoglobin A1c; SD, standard deviation; TAR, time above range (> 180 mg/dL; >10.0 mmol/L); TBR, time below range (< 70 mg/dL; <3.9 mmol/L); TIR, time in range (70–180 mg/dL; 3.9–10.0 mmol/L).

Similarly, individuals with higher HbA1c values at baseline showed greater reductions in HbA1c after AID initiation (Fig. 1b). A baseline HbA1c value of 6.7% (50 mmol/mol), 7.6% (60 mmol/mol), and 8.6% (70 mmol/mol) was associated with a mean HbA1c reduction of 0.3% (95% CI 0.2, 0.4), 0.8% (0.6, 0.9), and 1.3% (1.2, 1.4), equivalent to 2.9 mmol/mol (1.8, 4.1), 8.2 mmol/mol (6.8, 9.5), and 14.3 mmol/mol (12.9, 15.8), respectively (*P* < 0.001).

**Fig. 2:**
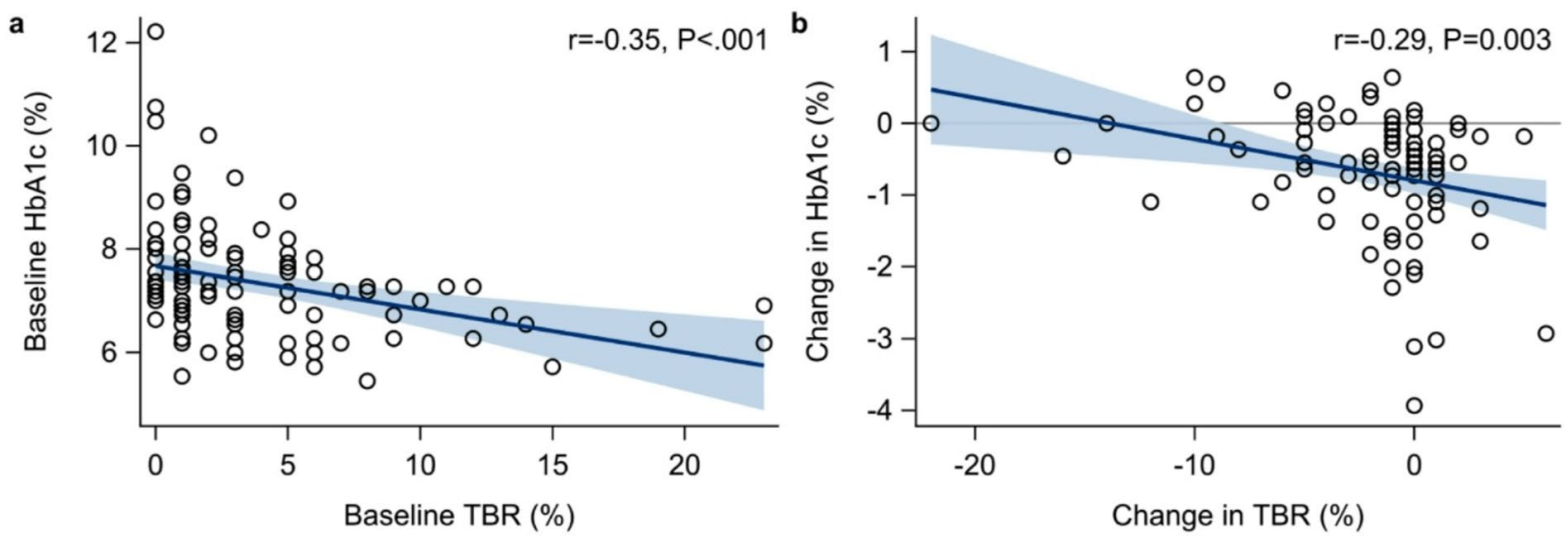
Association between below range (TBR; <3.9 mmol/L, < 70 mg/dL) and HbA1c: (**a**) baseline values and (**b**) changes after initiation of automated insulin delivery (AID). Points represent observed values. The blue line shows the fitted linear regression line, and the shaded band the corresponding 95% confidence interval for the trend line. r is the Pearson correlation coefficient.

Individuals with higher HbA1c had less TBR before starting AID (*r = −* 0.35 [− 0.50, − 0.16], *P* < 0.001; Fig. 2a). Those who experienced greater reductions in HbA1c after AID initiation showed smaller reductions in TBR and vice versa (*r* = − 0.29 [− 0.46, − 0.10], *P* = 0.003; Fig. 2b). In contrast, individuals with higher TBR at baseline had larger reductions in TBR following AID initiation (*r* = − 0.91 [− 0.94, − 0.88], *P* < 0.001).

No statistically significant differences in improvements of TIR, TAR, TBR, HbA1c, mean glucose level or glycemic variability (SD and CV) were observed in relation to prior insulin delivery modality (MDI vs. CSII), sex, diabetes duration, smoking status, or BMI (Table 2; Supplementary Tables 1–3).

### Subgroup analyses by AID system

Among users of the MiniMed 780G system, older age at initiation was associated with greater improvements in TIR (*r* = 0.51 [0.17, 0.74], *P* = 0.005) and mean glucose (*r* = − 0.52 [− 0.75, − 0.18], *P = *0.004). No significant correlations between age at initiation of AID and glycemic outcomes were found in the Tandem Control IQ group after adjusting for sex, BMI, and diabetes duration (Table 3).

**Table 3:** Correlation between age and changes in glycemic outcomes after AID initiation, stratified by AID system and adjusted for relevant baseline covariates.

	Tandem Control IQ (*n* = 92)	MiniMed 780G (*n* = 50)
Change in TIR	0.06 (− 0.21, 0.32) *P* = 0.66	0.51 (0.17, 0.74) *P* = 0.005
Change in HbA1c	0.13 (− 0.12, 0.36) *P* = 0.32	0.03 (− 0.33, 0.37) *P* = 0.89
Change in TAR	−0.09 (− 0.35, 0.18) *P* = 0.50	−0.39 (− 0.70, 0.04) *P* = 0.075
Change in TBR	−0.26 (− 0.49, 0.01) *P* = 0.054	0.20 (− 0.19, 0.54) *P* = 0.31
Change in mean glucose	−0.02 (− 0.27, 0.24) *P* = 0.89	−0.52 (− 0.75, − 0.18) *P* = 0.004
Change in SD of glucose values	−0.14 (− 0.39, 0.13) *P* = 0.32	−0.33 (− 0.67, 0.11) *P* = 0.14
Change in CV of glucose values	−0.37 (− 0.66, 0.03) *P* = 0.066	0.13 (− 0.48, 0.65) *P* = 0.69

Values are partial Pearson correlation coefficients with 95% confidence intervals (CI), adjusted for baseline values of the respective outcome and for sex, body mass index, and diabetes duration.

CI, confidence interval; HbA1c, hemoglobin A1c; SD, standard deviation; TAR, time above range (> 180 mg/dL; >10.0 mmol/L); TBR, time below range (< 70 mg/dL; <3.9 mmol/L); TIR, time in range (70–180 mg/dL; 3.9–10.0 mmol/L).

### Adverse events

Skin reactions caused by adhesives used in sensors or infusion sets were reported more frequently by women (41%) than by men (26%), although the difference did not reach statistical significance (proportion difference 15% [95% CI − 0.5, 31], *P* = 0.057). No other baseline characteristics were associated with the reporting of skin reactions (Supplementary Table 4). As reported in the original study^13^, four cases of severe hypoglycemia occurred in four participants related to physical activity and/or manual insulin dosing by the user. No events of ketoacidosis occurred during the follow-up period.

## Discussion

In this real-world cohort of adults with T1D, the greatest improvements in TIR and HbA1c occurred in individuals with the poorest glycemic control at baseline, irrespective of prior insulin delivery modality. Significant reductions in HbA1c were also observed among participants with baseline HbA1c levels closer to target, who additionally exhibited the greatest decreases in TBR. Older age at initiation of MiniMed 780G was associated with greater improvements in TIR.

Poor glycemic control as a predictor for greater improvements in HbA1c and TIR by AID system use aligns with the results from previous studies^6,11,14,15^. As previously reported from this cohort^13^, the improvements in glucose control were achieved without a concomitant increase in hypoglycemia. Among individuals with a baseline HbA1c of 70 mmol/mol, the initiation of AID was associated with an average HbA1c reduction of 14 mmol/mol. Reducing HbA1c levels in this subgroup of individuals is a clinical priority as they face an increased risk for microvascular complications and cardiovascular disease^16–18^.

Clinically meaningful reductions in HbA1c levels were also observed in adults with values closer to the recommended treatment target (< 7.0%, < 53 mmol/mol). This subgroup of individuals had greater TBR at baseline and exhibited the largest reductions in TBR following AID initiation. The value of AID in reducing hypoglycemic burden was emphasized by a recent randomized controlled trial^19^, comparing the Tandem Control IQ system to sensor-augmented pump therapy in reducing TBR in adults at high risk for hypoglycemia. The AID system was found to be superior already at 12 weeks of use. Reducing the hypoglycemic burden is a key clinical objective, as severe hypoglycemia may trigger fear of future hypoglycemic events, increase diabetes-related distress, and reduce well-being in adults living with T1D^20^. This in turn may prompt avoidance behaviors, including the maintenance of elevated glucose levels to reduce perceived hypoglycemic risk.

Previous MDI users achieved similar improvements in glucose control as individuals with prior pump experience, supporting the applicability of AID systems regardless of previous insulin delivery method. Importantly, participants in this study received education and follow-up in accordance with routine clinical practice in Sweden, without additional support within the scope of the study. The adaptability of AID systems was demonstrated in one study by Lepore and colleagues^15^, who conducted a retrospective study assessing the effects of the MiniMed 780G system over a two-year period. Their study included 296 adults with T1D, of whom 23% were prior MDI users. TIR increased by 12.8% during the first three months with no difference in relation to previous insulin therapy modality.

The observed association between older age at baseline and greater improvements in TIR among users of MiniMed 780G could indicate the need for extended support to younger users. These results align with a few prior observational studies including only users of the MiniMed 780G system^11,12^. Potential reasons could be differences in lifestyle including more spontaneous activities and meal irregularities in younger users. These findings should however be interpreted with caution given the observational nature of the analysis.

Although substantial progress has been made in the development and optimization of diabetes technologies, skin reactions resulting from materials used in sensors and infusion sets remain an underexplored area, as emphasized in a recent review article^21^. In our study cohort, one third of participants reported experiencing skin reactions when using AID, with a predominance in women. No other baseline characteristics emerged as correlating with the reporting of skin reactions. Prior observational studies have reported similarly high rates, ranging from 34 to 63%, among users of CGM and CSII devices^22–24^. Skin reactions have also been reported as a contributing factor for discontinuation of device use^23,25^. More tolerable adhesives are therefore needed.

Strengths of the study include its observational design, reflecting real-world use of AID systems in routine clinical practice in Sweden. Additional strengths include the random selection of participants, comprehensive glycemic data before and after AID initiation, and a substantial follow-up period. The study also documented adverse events such as severe hypoglycemia and ketoacidosis, patient-reported outcomes on practical barriers such as skin reactions and included individuals who had discontinued AID use. In addition, the study was performed independently of manufacturers of AID systems.

Limitations of the study include the absence of a control group, which restricts causal interpretation. However, the extended period of glucose data prior to the initiation of AID, together with the rapid improvement in glucose control after AID initiation that was sustained over time, argues against factors other than use of AID for the observed improvements. The questionnaire used to capture patient-reported barriers in daily use of AID was not validated. In addition, skin reactions related to sensors and infusion set adhesives were self-reported and may therefore be subject to reporting bias. Finally, the identification of rare adverse events would require a larger study population.

## Conclusions

In this real-world cohort, switching to AID substantially reduced HbA1c in adults with T1D having markedly impaired glycemic control. Significant reductions in HbA1c were also seen in individuals with HbA1c closer to target who additionally reduced their time in hypoglycemia the most. Older age was associated with greater improvements in TIR among users of the MiniMed 780G system. In summary, the use of AID systems in routine clinical practice benefits a broad range of adults with T1D, supporting wider implementation in diabetes care.

## Supplementary Information

Below is the link to the electronic supplementary material.

Supplementary Material 1

## Data Availability

The datasets used and/or analyzed during the current study are available from the corresponding author on reasonable request and after legal agreements for data sharing has taken place.
