# Supplementary material for: Effects of automated insulin delivery systems on glucose control in subgroups of adults with type 1 diabetes in clinical practice over 2 years in Sweden
Source: Sci Rep. 2026 Mar 31;16:11001. doi: 10.1038/s41598-026-37158-x (PMC13043898; doi:10.1038/s41598-026-37158-x)
Supplement: Supplementary file 1 — Supplementary Material 1 [file 41598_2026_37158_MOESM1_ESM.docx]

This supplement has been provided by the authors to give readers additional information about their work. Supplement to:

Effects of automated insulin delivery systems on glucose control in subgroups of adults with type 1 diabetes in clinical practice over 2 years in Sweden
 **Authors:** Singh R^1,2^, Imberg H^1,3^, Seyed Ahmadi S^1,2^, Hallström S^1,2^, Jendle J^4^, Tengmark Bengt-Olov^5^, Folino A^6^, Ekström M^7^, Lind M^1-2,7^.

**Author affiliations**

1. Department of Molecular and Clinical Medicine, Sahlgrenska Academy, University of Gothenburg, Gothenburg, Sweden.
2. Department of Medicine, Geriatrics and Emergency Care, Sahlgrenska University Hospital/Östra Hospital, Region Västra Götaland, Sweden
3. Statistiska Konsultgruppen Sweden, Gothenburg, Sweden
4. School of Medical Science, Faculty of Medicine and Health, Örebro University, Örebro, Sweden
5. Citydiabetes, Stockholm, Sweden
6. Department of Medicine and Emergency, Sahlgrenska University Hospital/Mölndal Hospital, Gothenburg, Sweden
7. Department of Medicine, NU Hospital Group, Uddevalla, Sweden

**Supplementary** **Table 1**. Differences in glycemic outcomes after AID initiation, by sex, unadjusted and adjusted for relevant baseline covariates.

|  | Female (n=58) | Male (n=84) | Unadjusted mean difference (95% CI) | *P* | Adjusted mean difference (95% CI) | *P* |
| --- | --- | --- | --- | --- | --- | --- |
| Change in TIR (%) | 13.2 (11.9) | 15.6 (12.7) | −2.5 (−7.2, 2.3) | 0.30 | −1.8 (−5.2, 1.6) | 0.29 |
| Change in HbA1c (%) | −0.6 (0.7) | −0.7 (0.9) | 0.1 (−0.2, 0.3) | 0.69 | 0.1 (−0.1, 0.3) | 0.48 |
| Change in TAR (%) | −11.9 (12.2) | −13.1 (13.7) | 1.2 (−4.0, 6.4) | 0.65 | 0.9 (−2.8, 4.6) | 0.64 |
| Change in TBR (%) | −1.5 (2.8) | −2.7 (4.9) | 1.2 (−0.4, 2.8) | 0.15 | −0.4 (−1.1, 0.4) | 0.33 |
| Change in mean glucose (mg/dL) | −14.3 (22.2) | −16.1 (28.2) | 1.8 (−7.8, 11.4) | 0.71 | 2.9 (−2.8, 8.5) | 0.31 |
| Change in SD of glucose values (mg/dL) | −7.7 (10.7) | −9.1 (15.0) | 1.4 (−3.8, 6.6) | 0.60 | −0.5 (−5.3, 4.3) | 0.83 |
| Change in CV of glucose values (%) | −1.9 (4.6) | −3.8 (6.6) | 1.9 (−1.2, 5.0) | 0.23 | 0.73 (−2.20, 3.66) | 0.62 |
| Descriptive data are presented as mean (SD).  Statistical analyses were conducted using two-sample *t*-tests in unadjusted analyses and analysis of covariance (ANCOVA) in adjusted analyses, adjusting for baseline values of the respective outcome as well as for age, body mass index, and diabetes duration.  **Abbreviations**: CI, confidence interval; CV, coefficient of variation; HbA1c, hemoglobin A1c; SD, standard deviation; TAR, time above range (>180 mg/dL; >10.0 mmol/L); TBR, time below range (<70 mg/dL; <3.9 mmol/L); TIR, time in range (70–180 mg/dL; 3.9–10.0 mmol/L). | | | | | | |

**Supplementary** **Table 2.** Differences in glycemic outcomes after AID initiation, by prior insulin delivery method, unadjusted and adjusted for relevant baseline covariates.

|  | CSII (n=104) | MDI (n=38) | Unadjusted mean difference (95% CI) | *P* | Adjusted mean difference (95% CI) | *P* |
| --- | --- | --- | --- | --- | --- | --- |
| Change in TIR (%) | 14.2 (12.4) | 15.5 (12.5) | −1.2 (−6.4, 3.9) | 0.64 | 2.1 (−1.7, 6.0) | 0.28 |
| Change in HbA1c (%) | −0.6 (0.8) | −0.7 (0.8) | 0.1 (−0.2, 0.4) | 0.53 | −0.0 (−0.3, 0.2) | 0.74 |
| Change in TAR (%) | −12.3 (12.8) | −13.2 (13.7) | 0.9 (−4.7, 6.5) | 0.76 | −2.3 (−6.5, 1.8) | 0.26 |
| Change in TBR (%) | −2.1 (4.6) | −2.4 (3.0) | 0.3 (−1.5, 2.0) | 0.76 | 0.6 (−0.3, 1.4) | 0.17 |
| Change in mean glucose (mg/dL) | −14.4 (25.8) | −18.0 (25.7) | 3.6 (−7.0, 14.2) | 0.50 | −3.8 (−10.3, 2.8) | 0.25 |
| Change in SD of glucose values (mg/dL) | −7.0 (13.0) | −12.5 (12.3) | 5.5 (−0.3, 11.3) | 0.061 | 2.1 (−3.4, 7.6) | 0.45 |
| Change in CV of glucose values (%) | −2.0 (5.8) | −5.1 (5.6) | 3.1 (−0.2, 6.4) | 0.067 | 2.4 (−0.6, 5.3) | 0.11 |
| Descriptive data are presented as mean (SD).  Statistical analyses were conducted using two-sample *t*-tests in unadjusted analyses and analysis of covariance (ANCOVA) in adjusted analyses, adjusting for baseline values of the respective outcome as well as for age, sex, body mass index, and diabetes duration. **Abbreviations**: CI, confidence interval; CSII, continuous subcutaneous insulin infusion; CV, coefficient of variation; HbA1c, hemoglobin A1c; MDI, multiple daily injections; SD, standard deviation; TAR, time above range (>180 mg/dL; >10.0 mmol/L); TBR, time below range (<70 mg/dL; <3.9 mmol/L); TIR, time in range (70–180 mg/dL; 3.9–10.0 mmol/L). | | | | | |  |

**Supplementary** **Table 3.** Differences in glycemic outcomes after AID initiation, by smoking status, unadjusted and adjusted for relevant baseline covariates.

|  | Current or former smoker (n=30) | Never smoker (n=104) | Unadjusted mean difference (95% CI) | *P* | Adjusted mean difference (95% CI) | *P* |
| --- | --- | --- | --- | --- | --- | --- |
| Change in TIR (%) | 19.4 (12.4) | 13.6 (11.9) | 5.8 (0.3, 11.3) | 0.038 | 0.4 (−3.4, 4.1) | 0.84 |
| Change in HbA1c (%) | −0.8 (0.7) | −0.6 (0.8) | −0.2 (−0.6, 0.1) | 0.18 | 0.0 (−0.2, 0.3) | 0.77 |
| Change in TAR (%) | −17.5 (14.8) | −11.7 (12.2) | −5.8 (−12.1, 0.6) | 0.073 | 0.4 (−3.9, 4.6) | 0.87 |
| Change in TBR (%) | −2.6 (3.6) | −2.1 (4.3) | −0.5 (−2.5, 1.4) | 0.59 | −0.0 (−0.8, 0.8) | 0.96 |
| Change in mean glucose (mg/dL) | −22.3 (28.4) | −14.0 (24.8) | −8.3 (−20.1, 3.5) | 0.17 | 2.0 (−4.4, 8.4) | 0.53 |
| Change in SD of glucose values (mg/dL) | −9.4 (12.7) | −8.4 (13.3) | −1.0 (−7.5, 5.4) | 0.75 | 2.3 (−2.7, 7.4) | 0.36 |
| Change in CV of glucose values (%) | −2.5 (7.7) | −3.1 (5.5) | 0.6 (−3.4, 4.7) | 0.76 | 0.1 (−3.1, 3.2) | 0.97 |
| Descriptive data are presented as mean (SD).  Statistical analyses were conducted using two-sample *t*-tests in unadjusted analyses and analysis of covariance (ANCOVA) in adjusted analyses, adjusting for baseline values of the respective outcome as well as for age, sex, body mass index, and diabetes duration. **Abbreviations:** CI, confidence interval; CV, coefficient of variation; HbA1c, hemoglobin A1c; SD, standard deviation; TAR, time above range (>180 mg/dL; >10.0 mmol/L); TBR, time below range (<70 mg/dL; <3.9 mmol/L); TIR, time in range (70–180 mg/dL; 3.9–10.0 mmol/L). | | | | | |  |

**Supplementary** **Table 4.** Association between baseline characteristics and reporting of skin reactions.

|  | Odds ratio (95% CI) | | *P* |
| --- | --- | --- | --- |
| Age, per 10 years increase | | 0.84 (0.65, 1.08) | 0.17 |
| Female sex | | 1.99 (0.97, 4.09) | 0.057 |
| Diabetes duration, per 10 years increase | | 0.87 (0.65, 1.18) | 0.37 |
| BMI, per 5 kg/m^2^ increase | | 0.97 (0.63, 1.49) | 0.88 |
| Current or former smoker | | 0.94 (0.40, 2.25) | 0.90 |
| CSII vs MDI | | 0.89 (0.40, 1.98) | 0.78 |
| TIR, per 10 % increase | | 0.98 (0.78, 1.22) | 0.83 |
| TAR, per 10% increase | | 1.04 (0.83, 1.29) | 0.74 |
| TBR, per 5% increase | | 1.01 (0.66, 1.55) | 0.97 |
| HbA1c, per 1 % increase | | 1.14 (0.83, 1.58) | 0.42 |
| Mean glucose, per 10 mg/dL increase | | 1.00 (0.89, 1.12) | 0.99 |
| SD of glucose values, per 50% increase | | 1.00 (0.81, 1.23) | 0.99 |
| Statistical analyses were performed using univariable logistic regression, with one baseline characteristic at a time entered as the explanatory variable. Results are reported as odds ratios (ORs) for skin reactions per x units increase in the explanatory variables.  **Abbreviations:** BMI, body mass index; CI, confidence interval; CSII, continuous subcutaneous insulin infusion; HbA1c, hemoglobin A1c; MDI, multiple daily injections; SD, standard deviation; TAR, time above range (>180 mg/dL; >10.0 mmol/L); TBR, time below range (<70 mg/dL; <3.9 mmol/L); TIR, time in range (70–180 mg/dL; 3.9–10.0 mmol/L). | | | |
